# The Green Shield: How Pro-Environmental Advocacy Protects Employees from Supervisor Ostracism

**DOI:** 10.3390/bs16020196

**Published:** 2026-01-29

**Authors:** Dong Ju, Yan Tang, Shu Geng, Ruobing Lu, Weifeng Wang

**Affiliations:** 1 Business School, Beijing Normal University, Beijing 100875, China; dongju@bnu.edu.cn (D.J.); tangyan@mail.bnu.edu.cn (Y.T.); wangweifeng@mail.bnu.edu.cn (W.W.); 2 Peking University Education Foundation, Peking University, Beijing 100871, China

**Keywords:** green advocacy, supervisor ostracism, moral credits, signaling theory, proactive prevention

## Abstract

Supervisor ostracism represents a pervasive and detrimental workplace stressor, yet existing research has predominantly focused on reactive coping mechanisms, leaving a critical gap regarding how employees can proactively prevent such mistreatment. To address this problem, this study draws on signaling theory as an overarching framework—integrated with social exchange theory as a downstream mechanism—to propose that employees can actively construct a “moral shield” by engaging in green advocacy, a high-cost, self-transcendent behavior that signals intrinsic moral character. We tested our theoretical model using a multi-method design. Study 1, a scenario-based experiment with 146 supervisors, provided causal evidence that green advocacy leads supervisors to objectively grant interpersonal moral credits, which subsequently reduces their behavioral intentions to ostracize. Study 2, a three-wave time-lagged survey of 434 employees, complemented these findings by confirming that green advocacy is associated with employees’ perceived moral credits and reduced perceived ostracism in a field setting. Furthermore, we found that this signaling process is contingent upon the receiver’s interpretation: the protective effect of green advocacy is amplified when Supervisory Support for the Environment (SSE) is high. This research contributes to the literature by identifying a novel, behavior-based signaling strategy for averting social exclusion and validating the dual nature (granted vs. perceived) of moral credits in hierarchical interactions.

## 1. Introduction

Workplace ostracism—the experience of being ignored or excluded by others at work—is a painful and pervasive phenomenon with severe consequences for both individuals and organizations (Ferris et al., 2008; Howard et al., 2020). When the source of this exclusion is a supervisor, the negative impacts are often amplified due to the inherent power imbalance (Cheng, 2024; Jahanzeb et al., 2018). Supervisor ostracism can thwart an employee’s fundamental psychological needs, leading to diminished well-being, reduced performance, and increased turnover intentions (Liu et al., 2022; Sarwar et al., 2025).

While the literature has documented the dark side of supervisor ostracism, it has paid less attention to the proactive, non-confrontational strategies employees might use to prevent it. Given that direct confrontation or whistleblowing can carry significant personal risk (Desai & Kouchaki, 2017), how can an employee, without directly challenging authority, shape a supervisor’s perception to avoid becoming a target of social exclusion? This study explores a subtle yet powerful strategy: employee green advocacy.

To build our argument, we develop a theoretical model anchored in signaling theory (Connelly et al., 2011; Spence, 1973), while positioning social exchange theory (SET) (Blau, 1964) as a downstream mechanism. We propose that by actively promoting pro-environmental behaviors, employees can broadcast a powerful signal about their unobservable moral character, thereby insulating themselves from supervisor ostracism. Specifically, we argue that green advocacy acts as a potent signal that reduces the information asymmetry between an employee and their supervisor regarding the employee’s underlying qualities, such as high moral standards and a commitment to the collective good (Connelly et al., 2011). Unlike general helping behaviors, which may be attributed to instrumental motives (Bolino, 1999), green advocacy serves the greater good, making it a purer and more credible validator of self-transcendence and intrinsic morality (Taufik et al., 2015; Minson & Monin, 2012). Crucially, we adopte a generative signaling perspective to distinguish this process from a purely relational one. While high-quality relationships (e.g., LMX) may naturally facilitate voice, we argue for a distinct behavior-to-status construction pathway: rather than viewing green advocacy merely as a byproduct of pre-existing safety, we conceptualize it as a proactive signaling strategy used to construct a moral shield. 

By actively broadcasting this costly signal, employees generate moral credits (Lin et al., 2016) in the eyes of their supervisors. While the concept of moral credits originated in the literature on moral self-licensing to describe an intra-psychic process (Merritt et al., 2010; Monin & Miller, 2001), we extend this logic to the interpersonal domain. Drawing on the concept of moral reputation (Carnes et al., 2022) and moral capital (Godfrey, 2005), we propose that moral credits can also represent a reservoir of goodwill accumulated in the eyes of observers. In this study, we define moral credits not as a license for the employee to misbehave, but as a protective status granted by the supervisor in recognition of the employee’s prosocial signals.

In addition, we integrate social exchange theory as a downstream mechanism to explain how the interpretation of these signals (i.e., moral credits) predicts the supervisor-subordinate relational dynamic and reduces ostracism. According to social exchange theory, supervisors are motivated to maintain positive, reciprocal relationships with high-value employees (Cropanzano & Mitchell, 2005). Ostracizing an employee who has demonstrated their value through such prosocial actions would violate the norm of reciprocity. Therefore, the accumulation of moral credits is expected to reduce the likelihood of experiencing supervisor ostracism.

Furthermore, we contend that the efficacy of this signal is contingent on the receiver’s values. The meaning and value of a signal are not inherent but are interpreted by the receiver (Connelly et al., 2011, 2025; Taj, 2016). Thus, we propose that supervisory support for the environment (SSE) will moderate this process. When a supervisor personally values environmental sustainability, an employee’s green advocacy is a more salient and credible signal of positive character, amplifying the accumulation of moral credits and strengthening the indirect, protective effect against ostracism (Lindsey et al., 2017).

**Figure 1:**
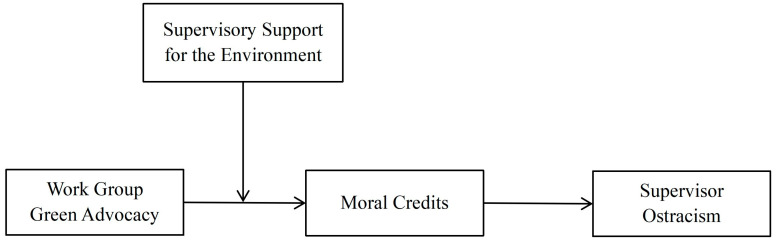
The theoretical model.

This research develops and examines a moderated mediation model (see Figure 1), which makes several key contributions. First, we advance the literature on workplace ostracism by identifying a proactive signaling strategy for prevention. Moving beyond the focus on reactive coping or victim characteristics (Liu et al., 2022), we demonstrate how employees can utilize green advocacy—a high-cost, self-transcendent behavior—to construct a protective moral shield. By grounding this in signaling theory, we explain how relatively benign advocacy behaviors function as high-efficiency signals of latent moral character, thereby altering supervisors’ exclusion decisions before they occur.

Second, we respond to calls for a more receiver-centric approach to signaling theory in management research (Connelly et al., 2011; Taj, 2016). Rather than assuming signals have fixed meanings, we identify supervisory support for the environment as a critical boundary condition that governs the signal decoding process. We demonstrate that SSE acts as a relevance filter, determining whether green advocacy is interpreted as a value-congruent signal (earning credits) or merely noise. This offers a theoretically precise explanation for when and why prosocial signals are effective.

Third, we extend and validate the concept of moral credits within the interpersonal domain. While traditional literature views moral credits as an intra-psychic licensing mechanism (Merritt et al., 2010), we theoretically position and empirically validate them as an interpersonal currency exchanged in hierarchical dyads. By utilizing a multi-method design—combining a scenario-based experiment (Study 1) that establishes causality with a time-lagged field survey (Study 2) that ensures ecological validity—we provide robust evidence that moral credits are not just self-perceived but are objectively granted by supervisors to shape social exchange dynamics.

## 2. Theoretical Framework and Hypothesis Development

We develop a two-stage theoretical model integrating signaling theory and social exchange theory to explain how employee green advocacy deters supervisor ostracism. Signaling theory serves as our overarching framework, explaining how employees communicate unobservable qualities through observable behaviors and how supervisors interpret these signals (Connelly et al., 2011; Spence, 1973). Social exchange theory then explains the downstream relational consequences: once supervisors perceive employees as possessing high moral character (signal reception), social exchange principles make ostracism of these valued partners relationally irrational and norm-violating (Blau, 1964; Cropanzano & Mitchell, 2005).

This integrated framework addresses a fundamental challenge in supervisor-subordinate relationships: information asymmetry regarding employee character and values (Connelly et al., 2011). Supervisors must make consequential decisions about resource allocation, developmental opportunities, and interpersonal treatment with incomplete information about employees’ underlying qualities. Employees, recognizing this asymmetry, have incentives to communicate positive qualities through observable behaviors—signals that supervisors can use to update their beliefs and shape subsequent treatment decisions.

### 2.1. Signaling High Moral Character Through Work Group Green Advocacy

Signaling theory is fundamentally concerned with reducing information asymmetry between two parties (Spence, 1973, 2002). In the workplace, supervisors (receivers) often have incomplete information about the unobservable qualities of their employees (signalers), such as their intrinsic moral character, prosocial orientation, or long-term commitment to the organization (Connelly et al., 2011). To reduce this asymmetry, employees can engage in observable actions or signals that credibly communicate these hidden positive attributes. One might question how a relatively specific and non-confrontational behavior like green advocacy can effectively deter a severe form of mistreatment like supervisor ostracism. We address this apparent asymmetry through the lens of signaling efficiency (Connelly et al., 2011; Spence, 1973). Drawing on Spence’s (1973) foundational insight—where modest educational investments signal large productivity differences—we argue that green advocacy functions as a high-leverage signal. While the act itself (e.g., advocating for recycling) may appear benign, it effectively broadcasts critical information about the employee’s latent moral character and prosocial orientation. This line of reasoning is inspired by research showing that subordinates can influence their superiors’ ethical behavior through “moral symbols” (Desai & Kouchaki, 2017), and we extend this logic to argue that prosocial behaviors serve a similar signaling function in the context of social inclusion.

For a signal to be effective, it must be both observable and costly, creating a “separating equilibrium” in which it is difficult for individuals who lack the underlying quality to credibly mimic the signal (Connelly et al., 2011; Spence, 1973). Employee green advocacy —defined as discretionary efforts to encourage pro-environmental actions among colleagues (Kim et al., 2017)—meets these criteria. It is an observable behavior that a supervisor can witness. More importantly, it is a costly signal. It requires time, effort, and social capital that extend beyond an employee’s formal job duties (Rodell & Lynch, 2016). Advocating for new initiatives can also carry social risks, such as being perceived as a nuisance by less-receptive colleagues. This inherent cost ensures the signal’s honesty; it is a behavior that an employee without a genuine commitment to the collective good would be unlikely to sustain (Connelly et al., 2025).

Research confirms that engaging in pro-environmental behaviors, even when required, can be perceived as acting against personal interests (e.g., costing more time) but serves the welfare of the whole, and is thus regarded as a moral behavior (Zhang et al., 2024). Specifically, engaging in green advocacy signals a strong moral character and a commitment to a collective good that transcends personal gain (Griskevicius et al., 2010; Vesely et al., 2020). It is important to note that signaling theory implies a reciprocal feedback loop between the sender and the receiver (Spence, 1973; Connelly et al., 2011). When supervisors (receivers) successfully decode the green advocacy signal and grant moral credits (as tested in Study 1), this change in cognitive evaluation is typically manifested in subtle relational cues and behavioral deference. Employees (senders), as active observers of their social environment, detect these cues and interpret them as an accumulation of moral capital.

This interpersonal moral credit granting represents the signal reception outcome in our signaling framework. The signal (green advocacy) communicates private information (moral character); the receiver (supervisor) observes and interprets the signal; and successful signal reception manifests as attributed moral credits—supervisors’ updated beliefs about employee moral standing.

**Hypothesis** **1:** *Work group green advocacy is positively related to moral credits.*

### 2.2. Moral Credits as a Buffer Against Supervisor Ostracism

The relationship between a supervisor and an employee can be understood through the lens of social exchange theory, which suggests that relationships are built upon a series of reciprocal exchanges of tangible and intangible resources (Blau, 1964). Supervisors, who control valuable resources such as information, support, and opportunities, are more likely to invest in and maintain positive relationships with employees they perceive as valuable and trustworthy exchange partners (Cropanzano & Mitchell, 2005).

Ostracism marks a breakdown in social exchange by withholding the social recognition and interaction that healthy work relationships need (Robinson et al., 2013). According to SET, moral credits accumulated through effective signaling serve as a relational currency. Supervisors, viewing these employees as valuable partners who have contributed to the collective morality, feel obligated to reciprocate with positive treatment—to refrain from negative treatment like ostracism—to maintain a balanced exchange relationship. To ostracize such an individual would be to sever ties with a high-value exchange partner, an act that is inconsistent with the principles of social exchange and potentially detrimental to the supervisor’s own team objectives. The accumulated moral credits thus serve as a buffer, making the employee a less likely target for the negative, exclusionary behavior of ostracism. Therefore, the moral credits earned through green advocacy should mediate the relationship between the employee’s signaling behavior and the supervisor’s exclusionary actions.

**Hypothesis** **2:** *Moral credits will mediate the relationship between work group green advocacy and supervisor ostracism.*

### 2.3. The Moderating Role of Supervisory Support for the Environment

Integrating the signaling framework, we propose that the translation of green advocacy into moral credits is strictly contingent upon the receiver’s interpretation process. Signaling theory posits that signal effectiveness depends not only on the sender but on the receiver’s calibration—specifically, whether the receiver deems the signal relevant to their own value system (Connelly et al., 2011; Taj, 2016). As Connelly et al. (2011) note, “receivers may apply weights to signals in accordance with preconceived notions about importance……” (p. 55). In our model, we conceptualize supervisory support for the environment (SSE) as a critical “relevance filter” governing this decoding mechanism.

When a supervisor possesses high SSE, they view environmental contributions as strategically and morally significant (Lindsey et al., 2017). In this context, the supervisor possesses the necessary cognitive schema to accurately interpret the employee’s behavior. Consequently, the employee’s green advocacy is successfully decoded as a high-value signal of character and commitment. The alignment between the signal’s content and the receiver’s values amplifies the signal’s weight in the supervisor’s mental calculus, facilitating a robust positive attribution process and the rapid accumulation of moral credits (Branzei et al., 2004).

Conversely, when a supervisor has low SSE, they lack the specific interpretive schema to appreciate the signal. Under these conditions, the receiver may prioritize immediate task efficiency over environmental goals. Consequently, the same green advocacy behavior risks being processed as signal noise or merely a distraction from core duties (Sauer et al., 2010). Because the receiver fails to assign positive weight to the signal—or views it as disingenuous virtue signaling (Connelly et al., 2025)—the transmission of moral character is disrupted. In such a context, the employee’s costly efforts fail to translate into objective status, preventing the accrual of moral credits. Thus, SSE determines the effectiveness of the signal transmission.

**Hypothesis** **3:** *Supervisory support for the environment will moderate the positive association between work group green advocacy and moral credits, such that this association will be stronger when the supervisor has higher (vs. lower) support for the environment.*

Building on this logic, the moderating effect of supervisory support for the environment should extend to the entire indirect relationship. The central mechanism in our model is the conversion of green behavior into moral credits, which then protects against ostracism. If this conversion process (the first stage of mediation) is stronger when supervisory support is high, then the overall protective effect of green advocacy should also be stronger under this condition. That is, the indirect effect of green advocacy on reducing supervisor ostracism via moral credits will be conditional on the supervisor’s environmental values.

**Hypothesis** **4:** *Supervisory support for the environment will moderate the indirect effect between work group green advocacy and supervisor ostracism via moral credits, such that this indirect effect will be stronger when supervisory support for the environment is high.*

### 2.4. Overview of Studies

We tested our theoretical model using a multi-method approach comprising two studies: a preregistered experiment (Study 1) and a three-wave, time-lagged field survey (Study 2). Study 1 employed an experimental design with a supervisor sample to test the proposed theoretical model by manipulating employee green advocacy and examining supervisors’ evaluations of employees’ moral credits and their ostracism intentions under varying levels of supervisory support for the environment. Study 2 provided a replication and extension of these findings in a sample of full-time employees. Using a three-wave, time-lagged survey with a subordinate sample, we examined the same theoretical relationships and extended Study 1 by shifting the focal outcome from supervisors’ ostracism intentions to employees’ experienced workplace ostracism.

## 3. Study 1 Method

### 3.1. Participants

In Study 1, we recruited participants from North America and Europe through Prolific. Previous research has shown that Prolific is a reliable source of data, comparable with traditional survey or lab research (Palan & Schitter, 2018; Peer et al., 2017). Before data collection, we pre-registered our experimental design and analyses on AsPredicted.org (https://aspredicted.org/ui29b9.pdf, accessed on 14 December 2025). In addition, before data collection, we conducted a priori power analysis based on a medium effect size of *d* = 0.50 and a significance level of 0.05 using G* Power (Faul et al., 2007). Results indicated that 128 participants are needed to achieve a 0.8 power. We thus collected data from 151 participants to account for participants who may fail our attention check (see exclusion criteria below).

To be eligible for the study, participants were required to be supervisors (i.e., have at least one subordinate) in their organizations and to currently live in North America or Europe. Participants were paid GBP 0.50 for participation. We followed the suggestion of Meade and Craig (2012) to add some basic attention check items (e.g., “Please select ‘Strongly Disagree’”) in the survey and excluded 5 respondents who failed the attention check. The final sample included 146 supervisors. They were 36.3% women and 72.6% held a bachelor’s degree or above. The average age of the participants was 45.2 years old, and 66.44% had more than 7 years of management experience.

### 3.2. Procedure and Experimental Design

In both Study 1 and Study 2, we strictly adhered to the ethical guidelines prescribed by the Institutional Research Ethics Committee of the authors’ university and the Declaration of Helsinki (World Medical Association, 2013). Prior to data collection for both studies, participants received an informed-consent statement that explained the research objectives, the voluntary nature of participation, and the strict confidentiality of their responses. Proceeding to the questionnaire was taken as consent.

At the outset of the survey, participants’ supervisor support for the environment (SSE) was assessed. This measure captured the participants’ pre-existing managerial orientation toward environmental sustainability, serving as a critical individual difference variable before any experimental stimuli were introduced. Then, the participants were instructed to read and engage in a scenario in which they were required to play the role of a team leader. They were informed that they would be managing a team of several employees, including a subordinate named Jess, who has been with the team for three years.

Participants were randomly assigned to one of two conditions (see Appendix A for a detailed description of the scenario):

Green Advocacy Condition (*n* = 73): In this scenario, Jess was depicted as an environmental champion. Beyond meeting core performance targets, Jess actively advocates for resource conservation, persuades colleagues to adopt waste-reduction habits, and proactively shares pollution-prevention strategies.

Control Condition (*n* = 73): In this scenario, Jess’s focus was confined to regular duties. While equally competent in core tasks, Jess shows limited initiative in environmental efforts, remains silent during sustainability discussions, and views ecological practices as secondary to immediate work goals.

Following the scenarios, participants completed measures of manipulation checks, moral credits, supervisor ostracism and reported their demographics.

### 3.3. Measure

Unless noted, all measures for the two studies were scaled using a five-point Likert-type scale ranging from 1 (strongly disagree) to 5 (strongly agree). All measures and items used across both studies can be found in Appendix B.

Moral Credits. Participants rated moral credits of the employee mentioned in the scenario (Jess) using the five-item measure developed by Lin et al. (2016). A sample item is “Jess earns credit for performing a morally laudable behavior” (α = 0.93).

Supervisor Ostracism Intentions. As we could not measure leaders’ actual ostracism in this vignette study, we followed previous research to measure supervisors’ ostracism intention (Ng et al., 2022; X. Xu et al., 2025; Hales et al., 2016). We measured it with Ng et al.’s (2022) ten-item scale which was adapted from the ostracism scale of Ferris et al.’s (2008) to fit our study context. Participants were asked to respond to sample items such as “I want to ignore Jess” (α = 0.98).

Supervisory Support for the Environment. To measure supervisory support for the environment, we adapted a six-item measure from Paillé et al. (2019). A sample item is “Encourage employees to attend environmental training” (α = 0.93).

Manipulation Check. After reading the text materials about Jess, the participants rated the employee’s green advocacy using the adapted three-item green advocacy scale of Kim et al. (2017). Participants rated how frequently Jess had engaged in such behaviors using a five-point Likert-type scale ranging from 1 (never) to 5 (always). A sample item is “Jess tries to convince the team members to reduce, reuse, and recycle office supplies in the workplace” (α = 0.97).

## 4. Study 1 Results

### 4.1. Manipulation Check

Participants in the employee green advocacy condition rated employee green advocacy to be significantly higher (*M* = 4.48, *SD* = 0.53) than those in the control condition (*M* = 1.66, *SD* = 0.88, *t*_[144]_ = −23.52, *p* < 0.001, *d* = −2.82), thereby indicating that the manipulation was successful.

### 4.2. Hypotheses Examination

**Figure 2:**
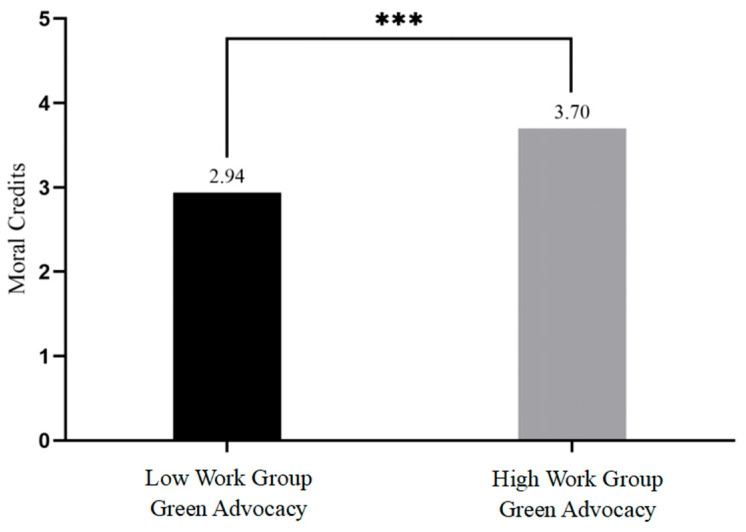
The effect of work group green advocacy on moral credits in study 1. *** *p* < 0.001.

Means, standard deviations, and correlations among study variables are reported in Table 1. Hypothesis 1 predicts that employee green advocacy is positively related to moral credits. The results (see Figure 2) revealed that supervisors assigned to the employee green advocacy condition reported significantly higher levels of perceived employee’s moral credits (*M* = 3.70, *SD* = 0.85) compared with those assigned to the control condition (*M* = 2.94, *SD* = 0.87; *t*_[144]_ = −5.37, *p* < 0.001, *d* = −0.77). Thus, Hypothesis 1 was supported.

We applied the PROCESS v4.2 macro by Hayes (2017) to conduct the proposed mediation, moderation, and moderated mediation analyses. The results of the regression analyses are shown in Table 2. All reported 95% confidence intervals (CIs) were calculated using a bias-corrected bootstrapping method with 5000 bootstrap samples.

The regression results (see Model 1 and 2 in Table 2) revealed that employee green advocacy was positively related to moral credits (*b* = 0.79, *p* < 0.001) and that moral credits was negatively related to supervisor ostracism intention (*b* = −0.33, *p* < 0.001). We used the bootstrapping procedure (Preacher & Hayes, 2008) with 5000 bootstrap samples to test the indirect effect. The results showed that the indirect effect of employee green advocacy on supervisor ostracism via moral credits was significant (effect = −0.26, 95% CI = [−0.48, −0.11]), supporting Hypothesis 2.

**Figure 3:**
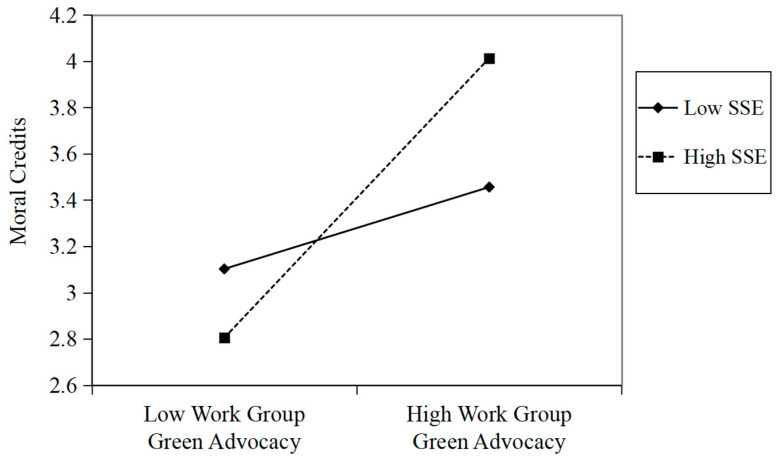
The moderating effect of SSE on the relationship between work group green advocacy and moral credits in study 1. Note. SSE = supervisory support for the environment.

Hypothesis 3 proposes that supervisory support for the environment moderates the relationship between employee green advocacy and supervisor ostracism such that the relationship is stronger when the supervisor has higher (vs. lower) support for the environment. The regression results of (see Model 2 in Table 2) revealed that employee green advocacy and supervisory support for the environment interacted to predict moral credits (*b* = 0.53, *p* < 0.01). We plotted this interaction (see Figure 3) and conducted a simple slopes analysis, which revealed a positive relationship between employee green advocacy and moral credits for high supervisory support for the environment (*b* = 1.21, *p* < 0.001) but not for low supervisory support for the environment (*b* = 0.35, *p* = 0.08). Thus, Hypothesis 3 was supported.

Hypothesis 4 posits that supervisory support for the environment will moderate the indirect effect between work group green advocacy and supervisor ostracism via moral credits, such that this indirect effect will be stronger when supervisory support for the environment is high. The results of bootstrapping analysis showed that the indirect effect was stronger for high supervisory support for the environment (effect = −0.41, 95% CI = [−0.78, −0.16]) than for low supervisory support for the environment (effect = −0.12, 95% CI = [−0.28, 0.02]) and that the index of moderated mediation (Hayes, 2015) was significant (index = −0.18, 95% CI = [−0.42, −0.02]). Thus, Hypothesis 4 was supported.

## 5. Study 2 Method

### 5.1. Procedure and Participants

In Study 2, we recruited our participants through Prolific following many previous studies (e.g., Ni et al., 2024; Venkataramani et al., 2024). To be eligible, participants were required to work full-time and have a direct supervisor, which was achieved through the use of Prolific’s screening tool. Data were collected from a diverse population to increase the heterogeneity of the participants, which enhances generalization of the research findings. In addition, we added attention-check questions in our surveys (e.g., “Please choose Strongly Disagree.”). To relieve the concern about common method bias, the study had a three-wave design with a seven-day time lag between each wave. We paid participants GBP 0.94 for the Time 1 survey, GBP 0.95 for the Time 2 survey, and GBP 1.00 for the Time 3 survey.

At Time 1, we asked participants to rate work group green advocacy and supervisory support for the environment. We received 600 complete responses, of which 573 passed attention-check questions. At Time 2, we asked participants to rate moral credits. Among those 573 participants who completed the Time 1 survey, 556 participants provided complete responses to the Time 2 survey. At Time 3, we asked participants to rate supervisor ostracism and to provide demographic information. Of these, 509 completed the Time 3 survey.

The data from three time points were matched using the participants’ Prolific IDs. After excluding missing data and removing responses from duplicate IP addresses (as some participants may have multiple Prolific accounts and participated in the survey multiple times), we obtained 434 matched and completed responses across three waves, yielding a response rate of 72.33%.

Among the participants, 50.5% were men and 49.5% were women; 86.2% held a bachelor’s degree or above. Their average age was 36.57 years (*SD* = 11.95), and their average organizational tenure was 7.27 years (*SD* = 6.11).

### 5.2. Measurement

Unless otherwise noted, participants rated all items on a 5-point Likert-type scale from 1 (strongly disagree) to 5 (strongly agree).

Work Group Green Advocacy. We measured employee’s work group green advocacy using the three-item scale developed by Kim et al. (2017). Participants indicated how frequently they would engage in such behaviors using a five-point Likert-type scale ranging from 1 (never) to 5 (always). A sample item is “I try to convince my group members to reduce, reuse, and recycle office supplies in the workplace” (α = 0.90).

Supervisory Support for the Environment. To measure supervisory support for the environment, we adapted a six-item measure from Paillé et al. (2019). A sample item is “My supervisor encourages environmental initiatives” (α = 0.96).

Moral Credits. Moral credits was measured using a five-item scale adapted from Lin et al. (2016). Participants were asked how much they agree with statements such as “I earned credit for performing a morally laudable behavior” and “Acting good built up my account of moral credits” (α = 0.93).

Supervisor Ostracism. We measured supervisor ostracism with Wu et al.’s (2015) ten-item scale which was adapted from the ostracism scale of Ferris et al. (2008) to fit our study context. Participants were asked to respond to sample items such as “My leader avoided me at work” and “My leader ignored me at work” (α = 0.95).

Control Variables. Gender represents a pertinent factor that might influence how people perceive and react to ostracism (Huang & Yuan, 2024). Older people (Pharo et al., 2011) and longer tenured employees (E. Xu et al., 2017) also are better at coping with ostracism. Therefore, we controlled participants’ gender, age, education and organizational tenure, which can influence how people react to mistreatment in organizations (E. Xu et al., 2017).

## 6. Study 2 Results

### 6.1. Measurement Model Validation

We conducted confirmatory factor analyses (CFA) using Mplus 8.3 (Muthén & Muthén, 1998–2017) to conduct a set of confirmatory factor analyses to examine the distinctiveness of the measures before testing the hypotheses. As presented in Table 3, our CFA results showed that the hypothesized four-factor model fitted better than alternative models (χ^2^ = 605.67, CFI = 0.96, TLI = 0.96, RMSEA = 0.06, SRMR = 0.04), showed that our theoretical model fits the data better than the other models.

Because Study 2 variables were collected from the same source, we assessed the potential impact of common method variance (CMV) using an unmeasured latent method factor approach (Podsakoff et al., 2003). Specifically, we compared the baseline four-factor measurement model to a model that included an orthogonal latent method factor loading on all items. The method-factor model showed a statistically significant improvement in fit, Δχ^2^(24) = 85.80, *p* < 0.001. However, two indicators suggested that the magnitude of method effects was not substantively problematic. First, the improvement in fit was small in practical terms (ΔCFI = 0.006, below the commonly used 0.05 threshold) (Jiang et al., 2018). Second, the method factor accounted for only 13.98% of the average item variance, indicating that CMV was present but not dominant. Taken together, these results suggest that CMV is unlikely to threaten the substantive conclusions in Study 2.

### 6.2. Descriptive Statistics and Simple Correlations

Table 4 presents the descriptive statistics, reliabilities, and correlations for the variables used in our study. The results showed that work group green advocacy was positively related to moral credits (r = 0.32, *p* < 0.001), which were negatively related to supervisor ostracism (r = −0.19, *p* < 0.001). These findings provide initial support for Hypotheses 1 and 2.

### 6.3. Hypotheses Examination

We applied the PROCESS v4.2 macro by Hayes (2017) to conduct the proposed mediation, moderation, and moderated mediation effects. The results of the regression analysis are shown in Table 5. All reported 95% confidence intervals (CIs) were calculated using a bias-corrected bootstrapping method with 5000 bootstrap samples.

To investigate whether work group green advocacy is positively related to moral credit and whether the effect of work group green advocacy on supervisor ostracism is mediated by moral credit, Model 4 in the SPSS PROCESS macro was employed. The results of Model 1 (Table 5) showed that work group green advocacy was positively related to moral credits (*b* = 0.26, *p* < 0.001), supporting Hypothesis 1.

Hypothesis 2 proposed that moral credits would mediate the relationship between work group green advocacy and supervisor ostracism. As can be seen from the results of Model 3 in Table 5, moral credits were negatively related to supervisor ostracism (*b* = −0.17, *p* < 0.001). The indirect effect was significant: effect = −0.045, 95% CI = [−0.077, −0.019], supporting Hypothesis 2.

**Figure 4:**
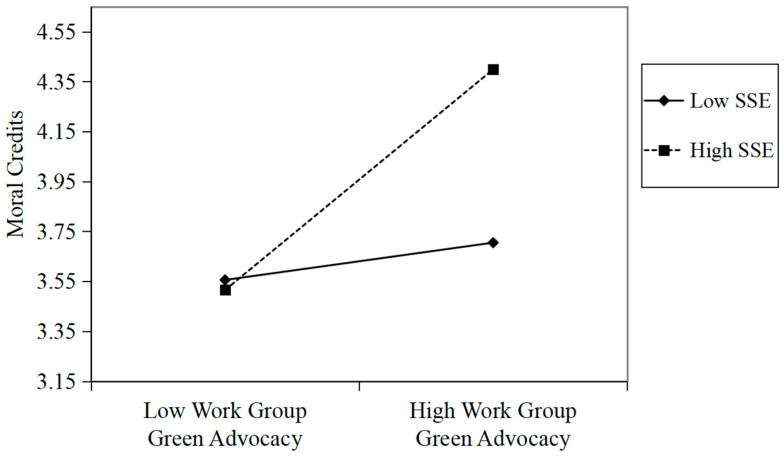
The moderating effect of SSE on the relationship between work group green advocacy and moral credits in study 2. Note. SSE = supervisory support for the environment.

To investigate the moderation effect (Hypothesis 3), Model 1 in the SPSS PROCESS macro was employed. Hypothesis 3 predicted that supervisory support for the environment would moderate the relationship between work group green advocacy and moral credits. As shown in Model 2 in Table 5, there was a significant interaction predicting moral credits (*b* = 0.17, *p* < 0.001). To further explain the form of the moderating effect, we conducted a simple slope analysis (Aiken & West, 1991). As presented in Figure 4, the simple slope test indicated that the relationship between work group green advocacy and moral credits was significant and positive when supervisory support for the environment was high (+1 SD) (*b* = 0.43, *p* < 0.001) but nonsignificant when it was low (−1 SD) (*b* = 0.07, n.s.). Additionally, the difference between these two slopes was significant (Δ*b* = 0.36, *p* < 0.001), supporting Hypothesis 3.

Finally, Model 7 in the SPSS PROCESS macro was used to test the moderation mediation effect. Hypothesis 4 proposed that supervisory support for the environment would moderate the indirect relationship of work group green advocacy on supervisor ostracism through moral credits. The results indicated that the indirect effect of work group green advocacy on supervisor ostracism was significant when supervisory support for the environment was high (indirect effect = −0.07, SE = 0.02, 95% CI = [−0.122, −0.030]) but not when supervisory support for the environment was low (indirect effect = −0.01, SE = 0.01, 95% CI = [−0.035, 0.013]). The index of moderated mediation was −0.03 (95% CI = [−0.052, −0.010]), indicating that these two indirect effects were significantly different from each other. Thus, Hypothesis 4 was supported.

## 7. Discussion

This study sought to understand how employees might proactively reduce their likelihood of being ostracized by their supervisors. Integrating signaling theory and social exchange theory, we demonstrated that engaging in green advocacy can serve as a protective strategy. Our findings indicate that employees who advocate for pro-environmental behaviors are perceived as having greater moral credits, which in turn diminishes their experience of supervisor ostracism. This mechanism, however, is contingent on the supervisor’s own values; the protective effect of green advocacy is only significant when the supervisor demonstrates high support for the environment.

### 7.1. Theoretical Contributions

This research makes several important theoretical contributions. First, we engage with the extensive literature on workplace ostracism, which has predominantly focused on consequences rather than prevention (Howard et al., 2020; Robinson et al., 2013). While Robinson et al. (2013) provided an influential theoretical model explaining why ostracism occurs (relational, instrumental, and punitive motives), less attention has been directed toward how targets might proactively deter such treatment. Our findings suggest that employees are not merely passive victims but can engage in strategic behaviors that shape supervisors’ perceptions and subsequent treatment decisions. This perspective contrasts with victim-focused research examining personal characteristics that predict ostracism (Liu et al., 2022) by emphasizing employee agency and proactive prevention strategies.

Second, our study advances a unified signaling-to-exchange framework to elucidate upward influence. We clarify that green advocacy functions as an informational catalyst: it first operates as a signal of moral character, which, upon successful reception, redefines the social exchange parameters between the dyad. By framing SET as the behavioral outcome of a prior signaling process, we provide a more coherent explanation of how unobservable character traits are translated into tangible interpersonal protection.

By validating this bottom-up signaling pathway, our research critically engages with emerging scholarly conversations regarding bidirectional influence in leader-follower relationships. While traditional research has predominantly focused on unidirectional leader effects—such as how transformational or ethical leadership shapes follower behavior (Kim et al., 2017; Robertson & Barling, 2013; Paillé et al., 2019)—our findings empirically demonstrate the reverse: subordinates actively shape supervisor perceptions and treatment decisions. This aligns with the relational leadership perspective (Uhl-Bien et al., 2014) and complements recent work on follower agency (Carton & Lucas, 2018; Wowak et al., 2016).

Third, we introduce and empirically validate the role of moral credits as a key psychological mechanism in an interpersonal context. While the concept originates from moral licensing research, which typically focuses on intra-individual processes (Merritt et al., 2010), our findings show that moral credits can be earned in the eyes of others and serve a protective function in social relationships, thereby broadening the applicability of this concept. Unlike previous research that relied solely on single-source data, our multi-method approach bridges the gap between objective causality (Study 1) and subjective perception (Study 2). We validate that green advocacy not only objectively shapes leader behavior but also alters the employee’s psychological experience of the workplace.

Finally, our findings engage with the ongoing scholarly debate regarding the relational value of prosocial behaviors. While Grant and colleagues have firmly established that prosocial acts generally enhance actors’ reputations and career outcomes (Grant & Patil, 2012; Grant & Mayer, 2009), emerging research reveals a more complex reality where such behaviors carry potential costs. For instance, Bolino et al. (2013) note that citizenship behaviors can lead to overload, while Rodell and Lynch (2016) demonstrate that noble acts like volunteering can be stigmatized rather than credited under certain conditions. We argue that this inconsistency stems from a limitation in how signaling mechanisms are traditionally viewed. As Taj (2016) critiques, signaling theory has often been applied too mechanistically, assuming signals have fixed meanings while neglecting how contextual factors shape signal reception. Similarly, Drover et al. (2018) advocate for a more cognitive approach that scrutinizes the receiver’s interpretation process. Bridging these perspectives, our findings contribute critical nuance to the debate: we demonstrate that the interpersonal benefits of prosocial behaviors are not universal but depend decisively on receiver characteristics. Specifically, green advocacy effectively protects against ostracism only when supervisors value the environment, but loses its signaling potency when they do not. This directly aligns with and empirically supports Connelly et al.’s (2025) recent call to examine how receiver attributes moderate signal effectiveness.

### 7.2. Practical Implications

First, this research identifies a proactive and non-confrontational strategy for building a positive reputation. In environments where direct communication about relationship issues may be difficult or risky, engaging in visible, prosocial behaviors like green advocacy can be an effective alternative. According to signaling theory, to be effective, a signal must be observed and perceived as reliable and honest (Drover et al., 2018). Employees should therefore choose advocacy actions that are visible to their supervisor, as only signals that capture attention can be processed and interpreted (Drover et al., 2018). They should also engage in these actions consistently over time; a single act may be dismissed, but a pattern of behavior sends a more credible signal of one’s underlying character because it demonstrates a costly and genuine commitment (Grimpe et al., 2019). Rather than vague statements, employees should translate their pro-environmental stance into concrete actions that managers are likely to perceive them as evidence of better performance and stronger organizational commitment (Cho & Jiang, 2022). Examples include researching and proposing a departmental paper-reduction plan, initiating a discussion about starting a composting program for office kitchen waste, organizing a carpooling or “bike-to-work” group, or creating a voluntary “green ideas” channel on a team communication platform to share knowledge.

Second, this research highlights the importance of recognizing the full value of employees’ prosocial behaviors. Supervisors should be trained to look beyond core task performance and appreciate that these actions can be signals of valuable, unobservable qualities like commitment, which in turn affect reward and promotion decisions (Cho & Jiang, 2022). Managers must also be acutely aware of how their own actions can reinforce or undermine employee signals. Sending mixed or negative signals—for instance, praising a green initiative but then denying resources—can damage the manager’s own credibility as a signaler and create a confusing and cynical environment (Gomulya & Mishina, 2017).

Furthermore, managers play a key role in shaping the “signaling environment” where employees’ actions are interpreted (Drover et al., 2018). They should publicly frame employees’ advocacy positively to prevent backlash or negative interpretations from peers, such as perceptions of favor-seeking (e.g., Campbell & Hahl, 2022; Inesi & Cable, 2015). This ensures the signal is interpreted as a contribution to the collective good rather than as individual grandstanding.

Third, this research offers a pathway to cultivate a more proactive and sustainable culture. Organizations can encourage voluntary green advocacy by creating a culture where such contributions are formally recognized and rewarded. This begins with hiring, as innovative firms already pay salary premiums to new hires with a background in advocacy, signaling that such experience is valued (Grimpe et al., 2019). To amplify the social benefits of these behaviors, organizations should create formal channels to increase their observability (Drover et al., 2018). HR can play a key role by establishing programs that recognize and celebrate these contributions, for instance, by featuring “Green Champions” in company-wide communications.

### 7.3. Limitations and Future Directions

This study has several limitations that open avenues for future research.

First, while we focused on green advocacy as a specific form of costly moral signaling, future research should examine whether other costly prosocial behaviors produce similar protective effects against ostracism. For instance, engaging in extensive helping behaviors, knowledge sharing that requires significant effort, or advocacy for other social causes (e.g., diversity initiatives, employee welfare programs) may also signal high moral character and serve as buffers against exclusion. Comparative studies examining the relative effectiveness of different types of prosocial signals would help build a more comprehensive theory of how employees can proactively manage their social standing and reduce ostracism risk through strategic prosocial behavior.

Second, our measurement of moral credits in Study 2 relies on employee self-reports to capture a construct that theoretically originates in the supervisor’s cognition. We acknowledge that relying on employees to assess the credits granted to them introduces potential self-report bias, as employees may lack direct access to their supervisors’ private attributions. That said, we sought to mitigate this limitation by conducting a supplementary experimental study (Study 1) that directly captured supervisor ratings, ensuring internal validity. Furthermore, research on meta-perception suggests that in established workplace relationships, employees generally possess accurate insights into how they are viewed by authority figures due to accumulated feedback and relational learning. Nevertheless, future research could benefit from collecting matched supervisor-subordinate dyadic data to more precisely capture the concordance between granted credits (supervisor perspective) and perceived credits (employee perspective).

Third, while this study links green advocacy to the prevention of a negative interpersonal outcome (ostracism), the signaling effects of such behaviors are likely broader. Future research should consider how various types of employee green behaviors might influence a wider range of organizational outcomes. Different green actions could signal different underlying qualities—for instance, resource conservation might signal conscientiousness, while proposing innovative green solutions could signal creativity and proactivity. These signals may, in turn, affect outcomes such as performance appraisals, promotion decisions, and organizational citizenship behaviors (OCB) (Rupp & Mallory, 2015). Investigating how these distinct signals influence a broader set of employee and organizational outcomes will deepen our understanding on the spillover effects of green behaviors in organizations.

## 8. Conclusions

In conclusion, this research identifies green advocacy as a novel, proactive signaling mechanism for averting workplace mistreatment. By validating the behavior-to-credit-to-status pathway across two studies, we provide a more complete understanding of how employees can navigate complex hierarchical relationships. However, the effectiveness of this strategy relies heavily on the receiver’s interpretation, necessitating a strategic fit between the employee’s signal and the supervisor’s values.

## Figures and Tables

**Table 1:** Descriptive statistics and correlations in study 1.

	*M*	*SD*	1	2	3
1. Employee green advocacy manipulation	0.50	0.50			
2. SSE	3.74	0.81	−0.11		
3. Moral credits	3.32	0.94	0.41 ***	0.08	
4. Supervisor ostracism	1.53	0.75	0.04	−0.14	−0.34 ***

*n* = 146; *n* = 73 in the employee green advocacy condition; *n* = 73 in the control condition; For the employee green advocacy manipulation, control condition = 0; employee green advocacy condition = 1; SSE = supervisory support for the environment. *** *p* < 0.001.

**Table 2:** Regression results for the predictors of moral credits and supervisor ostracism intention in study 1.

Variable	Moral Credits						Supervisor Ostracism		
	Model 1			Model 2			Model 3		
	*b*	*S.E.*	*t*	*b*	*S.E.*	*t*	*b*	*S.E.*	*t*
SSE	0.14	0.09	1.63	−0.18	0.14	−1.31	−0.08	0.07	−1.05
Employee green advocacy manipulation	0.79	0.14	5.55 ***	0.78	0.14	5.62 ***	0.30	0.13	2.36 *
Employee green advocacy manipulation × SSE				0.53	0.18	2.97 **			
Moral credits							−0.33	0.07	−4.92 ***
Constant	2.38	0.36	6.71 ***	2.95	0.10	29.98 ***	2.76	0.33	8.41 ***
*R* ^2^	0.18			0.23			0.16		
Δ*R*^2^				0.05 **					
*F*	15.93 ***			14.15 ***			9.16 ***		

*n* = 146; *n* = 73 in the employee green advocacy condition; *n* = 73 in the control condition; For the employee green advocacy manipulation, control condition = 0; employee green advocacy condition = 1; SSE = supervisory support for the environment. * *p* < 0.05. ** *p* < 0.01. *** *p* < 0.001.

**Table 3:** Confirmatory factor analyses results in study 2.

Model	χ^2^	*df*	Δχ^2^/Δ*df*	CFI	TLI	RMSEA	SRMR
Hypothesized 4-factor	605.67	246	—	0.96	0.96	0.06	0.04
3-factor (WGGA + SSE, MC, SO)	980.7	249	375.03/3 ***	0.93	0.92	0.08	0.05
2-factor (WGGA + SO + MC, SSE)	3354.82	251	2749.15/5 ***	0.69	0.66	0.17	0.22
1-factor	6107.22	252	5501.55/6 ***	0.41	0.35	0.23	0.27

WGGA = work group green advocacy, MC = moral credits, SO = supervisor ostracism, SSE = supervisory support for the environment; “+” means that the variables were combined. *** *p* < 0.001.

**Table 4:** Descriptive statistics and inter-correlations in study 2.

	*M*	*SD*	1	2	3	4	5	6	7
1. Gender	0.50	0.50							
2. Age	36.57	11.95	−0.01						
3. Education	3.22	1.00	0.12 *	0.01					
4. Organizational tenure	7.27	6.11	−0.03	0.62 ***	0.00				
5. Work group green advocacy	3.68	1.03	0.15 **	−0.11*	0.18 ***	−0.09			
6. SSE	3.64	1.07	0.19 ***	−0.18 ***	0.19 ***	−0.10 *	0.70 ***		
7. Moral credits	3.86	0.85	0.04	−0.06	0.06	−0.06	0.32 ***	0.31 ***	
8. Supervisor ostracism	1.56	0.72	−0.01	−0.06	−0.12 *	−0.04	−0.04	−0.19 ***	−0.19 ***

Note. *n* = 434. Gender: 0 = male; 1 = female. Education: 1 = High school diploma and below; 2 = Associate’s degree; 3 = Bachelor’s degree; 4 = Master’s degree; 5 = Doctoral degree. Tenure measured in years. SSE = supervisory support for the environment. * *p* < 0.05, ** *p* < 0.01, *** *p* < 0.001.

**Table 5:** Regression analyses results in study 2.

Variable	Moral Credits						Leader’s Ostracism		
	Model 1			Model 2			Model 3		
	*b*	*S.E.*	*t*	*b*	*S.E.*	*t*	*b*	*S.E.*	*t*
Gender	−0.02	0.08	−0.30	−0.03	0.08	−0.43	0.01	0.07	0.20
Age	−0.00	0.00	−0.26	0.00	0.00	0.23	−0.00	0.00	−1.10
Education	0.01	0.04	0.17	−0.01	0.04	−0.37	−0.08	0.03	−2.36 *
Organizational tenure	−0.00	0.01	−0.36	−0.00	0.01	−0.58	−0.00	0.01	−0.13
Work group green advocacy	0.26	0.04	6.80 ***	0.25	0.05	4.71 ***	0.02	0.04	0.65
Moral credits							−0.17	0.04	−4.07 ***
SSE				0.15	0.05	3.04 **			
Work group green advocacy × SSE				0.17	0.03	5.41 ***			
*R* ^2^	0.11			0.18			0.06		

Note. *n* = 434. 0 = male; 1 = female. SSE = supervisory support for the environment. Unstandardized regression coefficients are reported. * *p* < 0.05, ** *p* < 0.01, *** *p* < 0.001.

## Data Availability

The data presented in this study are available from the corresponding author upon request.

## Appendix A. Manipulations of Employee Green Advocacy in Study 1

Please read the following scenario carefully and play the role of a team leader. In your team, there are several employees, including Jess.

Green Advocacy Condition

Jess has been a member of your team for three years. He/She is regarded as a reliable and competent employee who consistently meets his/her core performance targets. In addition to his/her regular duties, Jess consistently champions environmental sustainability within the team.

He/She actively advocates for resource conservation, emphasizing extended reuse, and frequently persuades colleagues to cultivate waste-reduction habits. Jess demonstrates commitment through action, actively collaborating with colleagues to implement environmental practices and foster a positive ecological culture. Furthermore, in communication, Jess proactively shares strategies for pollution prevention in the workplace.

Control Condition

Jess has been a member of your team for three years. He/She is regarded as a reliable and competent employee who consistently meets his/her core performance targets. Confining his/her focus to his/her regular duties, Jess demonstrates limited initiative in the team’s environmental initiatives, preferring to focus his/her energy strictly on his/her assigned tasks.

Jess rarely engages in discussions regarding resource conservation or waste-reduction habit formation. When discussions regarding sustainability practices arise, he/she typically remains silent, viewing such considerations as secondary to his/her immediate work goals. Furthermore, Jess seldom offers insights or information concerning workplace pollution prevention.

## Appendix B. Scale Items Used in Studies 1 and 2

Work Group Green Advocacy (Study 1)

Jess tries to convince the team members to reduce, reuse, and recycle office supplies in the workplace.

Jess works with the team members to create a more environmentally friendly workplace.

Jess shares knowledge, information, and suggestions on workplace pollution prevention with other group members.

Supervisory Support for the Environment (Study 1)

Encourage environmental initiatives.

Encourage employees to attend environmental training.

Make sure that employees have environmental competences needed to do the jobs.

Openly engage in discussions around environmental topics.

Give complete and accurate information regarding environmental issues.

Involve employees in environmental problems solving.

Moral Credits (Study 1)

Jess earns credit for performing a morally laudable behavior.

Jess’s previous good deeds earn his/her credit as a moral person.

Acting good builds up Jess’s account of moral credits.

Each good deed Jess performed adds to his/her moral credit.

Acting in an ethical manner gives Jess a surplus of credit.

Supervisor Ostracism Intention (Study 1)

I want to ignore Jess.

I want to leave the area when Jess enters.

I don’t want to answer when Jess greets me.

I don’t want to sit with Jess.

I want to avoid Jess.

I don’t want to see Jess.

I don’t want Jess to join my conversation with other coworkers.

I don’t want to talk to Jess.

I want to treat Jess like he/she is invisible to me.

I don’t want to hang out with Jess for meals.

Work Group Green Advocacy (Study 2)

I try to convince my group members to reduce, reuse, and recycle office supplies in the workplace.

I work with my group members to create a more environmentally friendly workplace.

I share knowledge, information, and suggestions on workplace pollution prevention with other group members.

Supervisory Support for the Environment (Study 2)

My leader encourages environmental initiatives.

My leader encourages employees to attend environmental training.

My leader makes sure that employees have environmental competences needed to do the jobs.

My leader openly engages in discussions around environmental topics.

My leader gives complete and accurate information regarding environmental issues.

My leader involves employees in environmental problems solving.

Moral Credits (Study 2)

I earned credit for performing a morally laudable behavior.

My previous good deeds earned me credit as a moral person.

Acting good built up my account of moral credits.

Each good deed I performed added to my moral credit.

Acting in an ethical manner gave me a surplus of credit.

Supervisor Ostracism (Study 2)

My leader ignored me at work.

My leader left the area when I entered.

My greetings have gone unanswered by my leader.

My leader excluded me from having lunch together with him/her.

My leader avoided me at work.

I noticed my leader would not look at me at work.

My leader shut me out of the conversation.

My leader refused to talk to me at work.

My leader treated me as if I wasn’t there.

My leader did not invite me or ask me if I wanted anything when he or she went out for a tea break.
